# Uncovering a novel role of focal adhesion and interferon-gamma in cellular rejection of kidney allografts at single cell resolution

**DOI:** 10.3389/fimmu.2023.1139358

**Published:** 2023-03-31

**Authors:** Ahmad Halawi, Abdullah B. El Kurdi, Katherine A. Vernon, Zhabiz Solhjou, John Y. Choi, Anis J. Saad, Nour K. Younis, Rania Elfekih, Mostafa Tawfeek Mohammed, Christa A. Deban, Astrid Weins, Reza Abdi, Leonardo V. Riella, Sasha A. De Serres, Paolo Cravedi, Anna Greka, Pierre Khoueiry, Jamil R. Azzi

**Affiliations:** ^1^ Transplantation Research Center, Brigham and Women’s Hospital, Harvard Medical School, Boston, MA, United States; ^2^ Department of Biochemistry and Molecular Genetics, Faculty of Medicine, American University of Beirut, Beirut, Lebanon; ^3^ Q32 Bio, Waltham, MA, United States; ^4^ Scripps Clinic Medical Group, San Diego, CA, United States; ^5^ Clinical Pathology Department, Faculty of Medicine, Minia University, Minia, Egypt; ^6^ Department of Pathology, Brigham and Women’s Hospital and Harvard Medical School, Boston, MA, United States; ^7^ Department of Medicine, Division of Nephrology, Massachusetts General Hospital, Harvard Medical School, Boston, MA, United States; ^8^ Center for Transplantation Sciences, Department of Surgery, Massachusetts General Hospital, Boston, MA, United States; ^9^ Transplantation Unit, Renal Division, Department of Medicine, University Health Center of Quebec, Faculty of Medicine, Laval University, Québec, QC, Canada; ^10^ Translational Transplant Research Center, Icahn School of Medicine at Mount Sinai, New York, NY, United States; ^11^ The Broad Institute of Massachusetts Institute of Technology (MIT) and Harvard, Cambridge, MA, United States; ^12^ Department of Medicine, Brigham and Women’s Hospital, Boston, MA, United States

**Keywords:** graft rejection, kidney transplantation, single-cell analysis, cellular microenvironment, alloimmunity

## Abstract

**Background:**

Kidney transplant recipients are currently treated with nonspecific immunosuppressants that cause severe systemic side effects. Current immunosuppressants were developed based on their effect on T-cell activation rather than the underlying mechanisms driving alloimmune responses. Thus, understanding the role of the intragraft microenvironment will help us identify more directed therapies with lower side effects.

**Methods:**

To understand the role of the alloimmune response and the intragraft microenvironment in cellular rejection progression, we conducted a Single nucleus RNA sequencing (snRNA-seq) on one human non-rejecting kidney allograft sample, one borderline sample, and T-cell mediated rejection (TCMR) sample (Banff IIa). We studied the differential gene expression and enriched pathways in different conditions, in addition to ligand-receptor (L-R) interactions.

**Results:**

Pathway analysis of T-cells in borderline sample showed enrichment for allograft rejection pathway, suggesting that the borderline sample reflects an early rejection. Hence, this allows for studying the early stages of cellular rejection. Moreover, we showed that focal adhesion (FA), IFNg pathways, and endomucin (EMCN) were significantly upregulated in endothelial cell clusters (ECs) of borderline compared to ECs TCMR. Furthermore, we found that pericytes in TCMR seem to favor endothelial permeability compared to borderline. Similarly, T-cells interaction with ECs in borderline differs from TCMR by involving DAMPS-TLRs interactions.

**Conclusion:**

Our data revealed novel roles of T-cells, ECs, and pericytes in cellular rejection progression, providing new clues on the pathophysiology of allograft rejection.

## 1 Introduction

Kidney transplantation is the treatment of choice for patients with end-stage renal disease (ESRD). Transplant recipients are currently treated with nonspecific pharmacologic immunosuppressants with excellent short-term outcomes; however, chronic allograft rejection continues to be a challenge for long-term graft survival. Current immunosuppressive medications were developed based on their effect on T-cell activation, the dominant contributor to early rejection (1), rather than the underlying mechanisms driving alloimmune responses. Thus, understanding the alloimmune processes in the kidney microenvironment and the role of different cell populations will help us identify broad molecular targets to design more directed therapies with lower systemic side effects.

Single-cell RNA sequencing (scRNA-seq) techniques are promising for understanding the molecular fingerprints of individual cells within complex tissues and unraveling pathogenic mechanisms that can be targeted by novel immunomodulatory therapies. scRNA-seq has been successfully used in studying human kidney specimens (2, 3). Single nucleus RNA sequencing (snRNA-seq) differs from scRNA-seq in that the nuclei alone are isolated to study gene expression rather than the entire cell (4). Although the use of scRNA-seq offers more mRNA per cell to sequence, its use is limited due to its incompatibility with frozen archival material. Moreover, snRNA-seq was recently shown to be comparable gene detection to scRNA-seq in the adult kidney (5).

Cellular allograft rejection is characterized by immune cells, primary T-cells, and macrophage graft invasion with subsequent inflammation and tissue destruction (6). Based on the Banff classification of renal allograft rejection, T-cell mediated rejection (TCMR) is characterized by significant immune cell interstitial infiltration, tubulitis, and arteritis (7). Borderline rejection is defined as a condition with less severe inflammation than TCMR (7). Whether the borderline changes are an early rejection state that can inform us on the earlier pathways involved in the rejection process is still unclear (8). Apart from T-cells, the endothelial cells (ECs) serve as a unique barrier between the blood and the tissues, and are actively involved in cellular rejection through their early role in the activation and migration of alloreactive CD4+ and CD8+ T-cells (9). Pericytes are multi-functional mural cells of the microcirculation that wrap around the endothelial cells. Recently, pericytes were shown to play a role in T-cell modulation (10) and constituting together with the endothelium and its basement membrane a physical barrier to immune cells invasion (11, 12).

To characterize the complexity of the cellular rejection process at the molecular level, we studied the transcriptional signatures of T-cells, ECs, and pericytes in human non-rejection, borderline, and TCMR specimens using snRNA-seq. We found that T-cells in the borderline sample were enriched for allograft rejection signature, suggesting that borderline is an early cellular rejection process; however, ECs but not pericytes significantly differ between the borderline and TCMR samples. In particular, we found significant differences in the integrin signaling pathway (also known as focal adhesion pathway), response to interferon-gamma (IFN-gamma), and interactions with both T-cells and pericytes in ECs obtained from borderline versus TCMR samples. Our data provide new insights into the role of the endothelium, pericytes, and T-cells in the different phases of the cellular rejection process.

## 2 Materials and methods

### 2.1 Tissue collection and single nucleus dissociation, single-nucleus capture, library preparation, and sequencing

We performed snRNA sequencing of three human kidney allograft biopsies from non-rejection, borderline, and TCMR (IIa) patients. The three recipients were males. Recipients with non-rejection and TCMR received their grafts from living female donors aged 66 and 67. As for native kidney disease, the patient with non-rejection had renal cell carcinoma and hypertension, whereas patients with borderline and TCMR were diagnosed with hypertension and diabetes mellitus type 1, respectively (**Supplementary Table 1**). Frozen kidney biopsy specimens were obtained from our patients after appropriate consent and in accordance with MASS GENERAL BRIGHAM IRB and institutional guidelines. After first removing the surrounding optimal cutting temperature (OCT) embedding medium with PBS, a previously published protocol using salt Tris-based buffers was used to isolate single nuclei (13). 8,000 single nuclei were then loaded into each channel of the Chromium single cell 3’ chip (v3; 10x Genomics, Pleasanton, USA).

Single nuclei were partitioned into gel bead-in-emulsions (GEMs) and incubated to generate barcoded cDNA by reverse transcription. Barcoded cDNA was then amplified by PCR prior to library construction. Fragmentation, sample index, adaptor ligation, and PCR were used to generate libraries of paired-end constructs according to the manufacturer’s recommendations (10x Genomics, Pleasanton, USA). Libraries were pooled and sequenced using the Illumina HiSeq X system (San Diego, USA).

### 2.2 Single-nucleus RNA-seq data analysis

Demultiplexing and counting were performed using CellRanger 5.0. The generated raw, sparse matrices were subject to quality checks followed by removal of empty cells using emptyDrops function from ‘DropletUtils’ R package version ‘1.4.3’. All cells with less than 450 UMIs were considered empty cells and were filtered out. We then used Seurat version ‘3.2.2’ for normalizing, scaling, clustering, and annotation. First, genes expressed in more than three cells and cells that expressed more than 100 genes were kept for downstream analysis. Cells expressing more than 20% of mitochondrial counts were excluded. Additionally, we kept cells that express less than 3500, 4000, and 6000 genes for non-rejection, TCMR, and borderline rejection samples, respectively. Doublets/multiplets were removed using ‘DoubletFinder’ R package version ‘2.0.3’. The remaining cells used for integration were 773, 2790, and 6083 cells for non-rejection, TCMR, and borderline rejection, respectively.

### 2.3 Integrating samples and finding differentially expressed genes for endothelial cells in TCMR *vs*. borderline

The three analyzed samples were merged using the ‘merge’ function from Seurat package. Next, 2000 variable features were identified using ‘SelectIntegrationFeatures’ after running ‘FindVariableFeatures’ on each sample. ‘FindIntegrationAnchors’ and ‘IntegrateData’ functions were used to integrate the three samples. Non-linear dimensional reduction on scaled data was applied, followed by clustering using dimensions 1:16 and a resolution of 0.3. Differential expression analysis was performed between endothelial clusters and T cells clusters belonging to TCMR and borderline rejection using ‘FindMarkers’ function from Seurat, with ‘min.pct=0.25’ and ‘logfc.threshold=0’. Using ‘VlnPlot’ function, the expression of the integrin signaling pathway, VEGF, angiogenesis, interferon-gamma response, and antigen processing and presentation genes were plotted for all three samples. Clusters were plotted by applying ‘DimPlot’ function, using ‘umap’ as a reduction method. Marker genes for each cluster were identified using ‘FindAllMarkers’ function, with Wilcoxon rank-sum as a statistical test, with the parameters ‘min.pct=0.25’ and ‘logfc.threshold=0’. The annotation of clusters was made using marker genes based on literature search and confirmed using enrichR (14).

### 2.4 Gene sets enrichment analysis and pathways analysis

Functional analysis was performed on the differential expressed genes (DEG) between TCMR and borderline rejection endothelial clusters *via* Gene Sets Enrichment Analysis (GSEA) (15), using the Java GSEA implementation. We chose the hallmark gene sets that contain 50 gene sets to run the analysis. We also used Enrichr (14), an interactive and collaborative HTML5 gene list enrichment analysis tool, to run pathways analysis using PANTHER (16)database for the differentially expressed genes between the three endothelial clusters from the three samples and for the differentially expressed genes for T cells between borderline rejection and TCMR using MsigDB (17) and Hallmarks (18). A volcano plot and bar plots were generated using ‘ggplot2’ R package.

### 2.5 Ligand-receptor analysis

Ligand-Receptor (L-R) analysis was conducted between T cells, pericytes, and EC, in TCMR and borderline rejection. ‘SingleCellSignalR’ (19), a Bioconductor R package, was used to assess the interactions between the clusters. This package includes a curated database of ligand-receptor interactions called ‘LRdb,’ containing 3251 ligand-receptor pairs compiled from many databases. The top 40 interactions were shown between ligands and receptors in a circos plot.

## 3 Results

### 3.1 snRNA-seq identifies major renal populations

**Figure 1:**
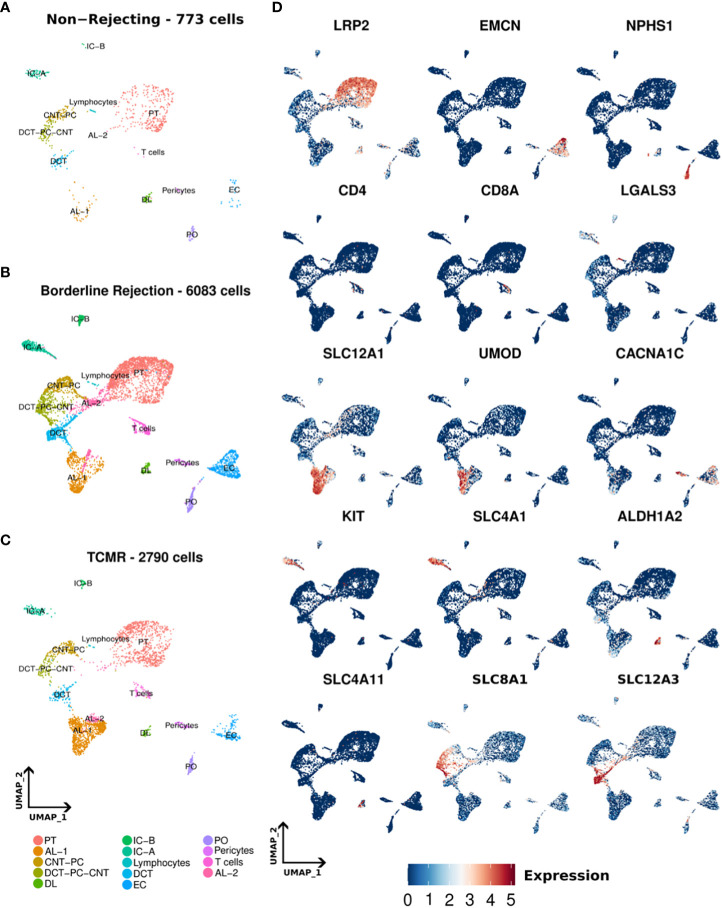
**(A–C)** Umaps of non-rejecting, borderline, and T-cell mediated rejection (TCMR) samples showing different clusters. **(D)** Umaps showing the expression of certain canonical markers used for cluster annotation. PT (Proximal tubules), AL-1 (Ascending loop of Henle-1), AL-2 (Ascending loop of Henle-2), CNT-PC (Connecting tubules-Principal cells), DCT-PC-CNT (Distal convoluted tubules-Principal cells- Connecting tubules), DL (Descending loop of Henle), IC-A (intercalated cells A), IC-B (intercalated cells-B), DCT (Distal convoluted tubules), EC (Endothelial cells), PO (podocytes).

We conducted snRNA-seq on one non-rejecting kidney allograft sample, one borderline change sample, and TCMR (IIa) sample as classified by Banff criteria (7). Following data processing, a total of 773, 6083, and 2790 nuclei passed quality filters in non-rejecting, borderline, and TCMR samples, respectively (Methods). We identified 14 clusters in normal, borderline, and TCMR samples (**Figures 1A–C**). We annotated our clusters using anchor genes previously described in the literature (2, 19–21) (**Figure 1D**; **Supplementary Tables 2, 3**). Overall, we identified the major kidney cell populations in addition to immune clusters. The kidney cell populations included epithelial, stromal, and endothelial clusters. In addition, we identified immune clusters, including T-cell and lymphocyte clusters, in the three samples. The lymphocyte cluster showed general markers of lymphocytes and did not express clear markers for immune subpopulations. Stromal nuclei were clustered as pericytes. Among the epithelial cells, we found podocytes (PO), epithelial cells of the proximal tubule (PT), ascending loop of Henle (AL) 1 and 2, descending loop of Henle (DL), distal convoluted tubule (DCT). Collecting ducts included intercalated cells A (IC-A) and B (IC-B), and transitory populations expressing genes of different adjacent cell types, connecting tubules (CNT), and the principal cells (PC) that we annotated as DCT-CNT-PC and CNT-PC. These clusters are distributed across all three samples, as shown in the UMAPs of **Figure 1**. Some anchor genes used for annotation are shown in **Figure 1D**.

### 3.2 T-cell cluster in borderline sample show enrichment for allograft rejection and interferon-gamma and alpha response pathways

**Figure 2:**
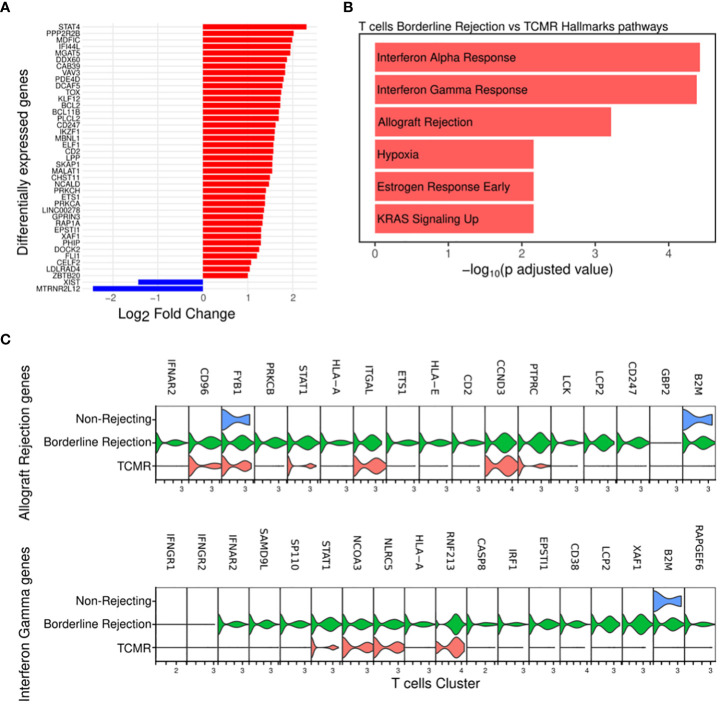
**(A)** Plot showing the differentially expressed genes between borderline (red) and TCMR (blue). **(B)** Pathway analysis of T-cell cluster in borderline compared to TCMR samples using Hallmark database showed enrichment for interferon-gamma and alpha responses and allograft rejection pathways **(C)** Violin plots showing the level expression of genes part of allograft rejection and interferon-gamma response pathways in three conditions.

Since T-cells, identified in both TCMR and borderline samples, are the main effectors of cellular rejection, we focused our initial efforts on pathway analysis in these clusters. We noted an increase in the percentage of T-cells in both borderline (4.4% of total cells) and TCMR (2.5% of total cells) compared to non-rejection, where it was 0.39% (**Supplementary Table 2**). We identified differentially expressed genes in borderline compared to TCMR (**Figure 2A**; **Supplementary Table 4**), and we performed pathway analysis. Interestingly, T-cell cluster in borderline sample showed enrichment for allograft rejection, interferon-gamma (IFN-gamma) response, and interferon-alpha (IFN-alpha) response pathways when using Hallmark datasets (18) (**Figure 2B**). The allograft rejection pathway includes a set of up-regulated genes identified in studies of solid organ rejection (22–26). The IFN-gamma response pathway includes genes up-regulated in response to IFN-gamma (27–30). IFN-gamma is a proinflammatory cytokine that plays a significant role in the polarization of th1 (31) and activation of cytotoxic T-cells (32). In allograft rejection, studies on IFN-gamma demonstrated two contrasting functions: protective in the early course and destructive late in disease progression (33). Hence, in borderline condition, T-cells seem to be activating pathways implicated in allograft rejection and responding to a high IFN-gamma and alpha milieu.

Then, we compared the level of expression of genes involved in enriched pathways. Results showed that the borderline rejection sample expresses more genes implicated in allograft rejection and IFN-gamma response than TCMR (**Figure 2C**). T-cells in TCMR downregulates B2M and HLA-A gene expression, which are involved in antigen processing and presentation. Genes implicated in the allograft rejection pathway appear uniformly up-regulated in borderline change. Of these genes, we notice PTPRC (CD45), LCK, and CD247 (CD3ζ), which are implicated in T-cell receptor signaling (34, 35).

Thus, T-cells show a transcriptional profile of allograft rejection in borderline with more broad gene expression, suggesting that the borderline sample reflects an early rejection state.

### 3.3 Integrin signaling pathway and interferon-gamma response are specifically downregulated in the endothelial cluster of TCMR

The endothelial cells are the first cells exposed to the recipient’s immune system and form an essential barrier for leukocyte transmigration. The transmigration is an active process that mandates the passage of immune cells from the vessel’s lumen to the graft tissue passing through the endothelium and the peri-endothelial structures (36).

**Figure 3:**
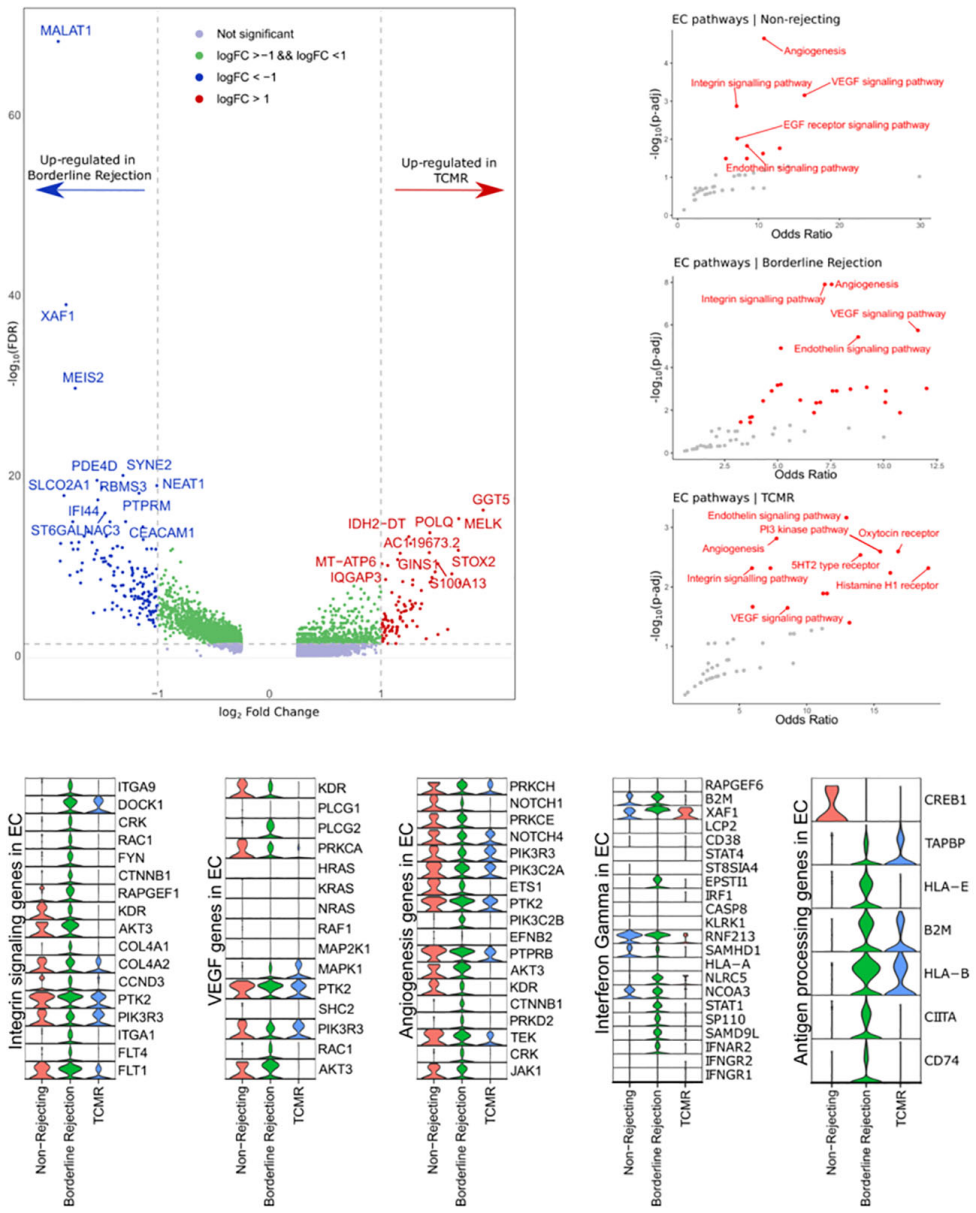
**(A)** Volcano plot showing the up-regulated genes in endothelial cell clusters (ECs) between borderline and TCMR. Blue and red dots and genes up-regulated in borderline and TCMR, respectively; green dots are genes with log-fold change (logFC) between 1 and -1; gray dots are genes that did not reach a significance level. **(B)** Scatter plots showing the top enriched pathways in non-rejection, borderline, and TCMR samples. **(C)** Violin plots showing the level expression of genes part of integrin signaling, VEGF, angiogenesis, interferon-gamma response, and antigen processing pathways in ECs clusters in the three conditions.

Since T-cells showed an active rejection phenotype in borderline, which is usually seen in TCMR, we wanted to investigate the properties of ECs in affecting the rejection process by identifying differentially expressed genes and enriched pathways. Differential gene expression analysis identified 283 and 77 genes up-regulated in borderline and TCMR samples, respectively (**Figure 3A** and **Supplementary Table 5**). The top 10 DEGs in borderline sample were: MALAT1, XAF1, IFI44L, EMCN, MEIS2, PBX1, SYNE2, PDE4D, NEAT1, and RBMS3. However, the top 10 DEGs in TCMR were: MTRNR2L12, XIST, PVT1, GGT5, MELK, POLQ, IDH2-DT, MT-ATP6, IQGAP3, and S100A13.

We used Enrichr (14) to identify enriched pathways with links to the identified up-regulated genes (**Figure 3B**). In ECs of borderline and normal samples, integrin signaling pathway, also known as focal adhesion (FA), angiogenesis, and VEGF signaling pathways, were strongly enriched with a high odds ratio and statistical significance (**Figure 3B**). In the TCMR ECs cluster, the endothelin signaling pathway showed a higher significance, and the integrin signaling pathway showed a lower odds ratio than other samples.

Next, we compared the gene expression level of integrin signaling, VEGF, and angiogenesis pathways between ECs clusters in the three samples (**Figure 3C**). ECs in the borderline sample express major genes implicated in the integrin signaling pathway. Nevertheless, ECs in non-rejection and TCMR showed down-regulation of many of these genes. Membranous proteins, such as ITGA1, ITGA9, and FLT4, are expressed in ECs of the borderline sample. KDR, known as VEGFR2, shows expression in non-rejection and borderline samples but not in TCMR. Following the interaction of integrins (ITGs) with extracellular matrix (ECM) molecules such as fibronectin, subsequent signaling cascade leads to cell motility, proliferation, or survival based on multiple interactions of activated networks within the integrin signaling pathway. The ITG-FAK-RAC network is part of the integrin signaling pathway implicated in adhesion and motility (37–39). Its genes include DOCK1, CRK, RAC1, and ITGs, exclusively up-regulated in ECs of the borderline sample (except for DOCK1, which is also up-regulated in ECs of borderline and TCMR samples; **Figure 3C**). Therefore, a specific network associated with endothelial adhesion is up-regulated in the borderline sample that is unseen in the TCMR sample.

Moreover, we used GSEA to analyze DEGs between borderline and TCMR. Hallmarks of IFN-alpha and -gamma response were up-regulated in ECs of borderline and down-regulated in TCMR and non-rejection (**Supplementary Figure 1**). Expression analysis of IFN-gamma response genes reveals minimal expression in ECs of non-rejection and TCMR samples compared to a broad expression in the borderline sample (**Figure 3C**). Genes that showed high expression in ECs of both TCMR and borderline were: B2M, XAF1, RNF213, and NCOA3. Genes predominantly up-regulated in ECs of the borderline sample were: IFNAR2, SAMD9L, STAT1, NLRC5, SP110, and ESPTI1. These genes are part of the signaling pathway of IFN-gamma once it activates its receptor on the ECs. IFNGR1/2 did not show expression in ECs of the three samples, possibly due to the low mRNA level in the nucleus.

Since IFN-gamma is well known to stimulate the antigen processing and presentation pathway in ECs, we hypothesized that genes implicated in antigen processing and presentation are up-regulated in ECs of the borderline sample compared to TCMR and non-rejection samples. Indeed, ECs in the borderline sample up-regulated essential antigen processing and presentation genes such as CD74, CIITA, HLA-E, TAPBP, and HLA-B, where TCMR showed only up-regulation of HLA-B, B2M, and TAPBP (**Figure 3C**).

Hence, we conclude that the response to IFN-gamma is significantly suppressed in ECs in the TCMR sample, including antigen processing and presentation.

### 3.4 Ligand-receptor analysis reveals interactions of endothelial cells with T-cells and pericytes in borderline and TCMR

**Figure 4:**
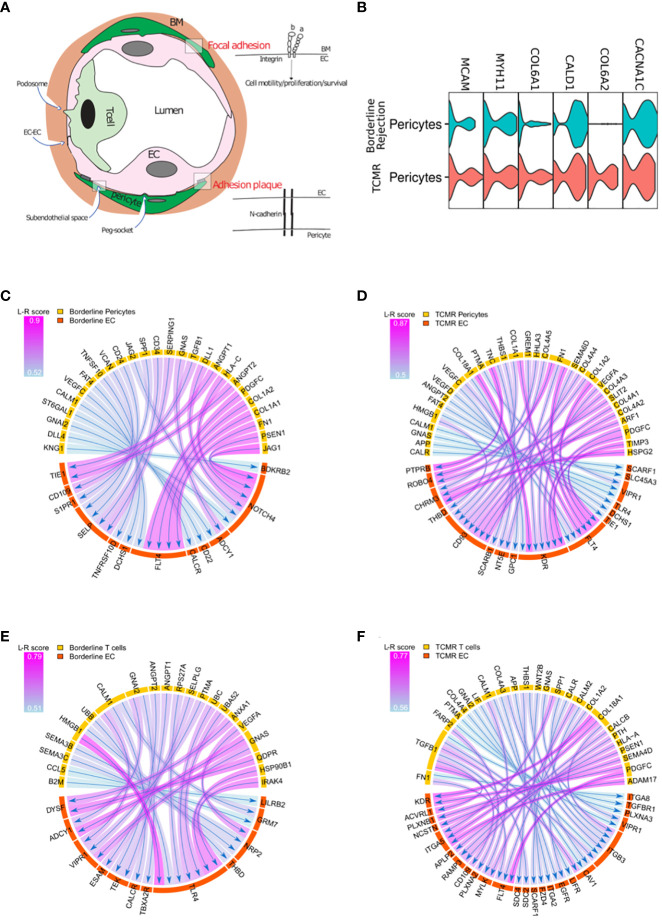
**(A)** T-cells, endothelial cells (EC), and pericytes interactions. Focal adhesion represents the interaction between ECs and the surrounding basement membrane (BM); adhesion plaque is the interaction between ECs and pericytes. **(B)** Violin plot showing canonical markers expressed by pericyte clusters in borderline and TCMR. **(C, D)** Circos plot showing the ligand-receptor interactions between pericytes in yellow and endothelial cells in orange in borderline and TCMR; the heads of arrows are toward the receptor side; the halo color around the arrow represents the strength of this interaction as per L-R score. **(E, F)** Circos plot showing the ligand-receptor interactions between T-cells in yellow and endothelial cells in orange in borderline and TCMR; the heads of arrows are toward the receptor side; the halo color around the arrow represents the strength of this interaction as per L-R score.

Based on the difference in endothelial transcriptional profile between borderline and TCMR, especially genes related to integrin signaling and angiogenesis, we hypothesized that ECs in TCMR and borderline samples receive different signals from surrounding cells which translate to different transcriptional profiles. In the abluminal side of the vessels, ECs are attached to the basement membrane (BM) *via* integrin-mediated focal adhesion (40) and to pericytes *via* N-cadherin interaction (41) (**Figure 4A**). After the transendothelial migration, leukocytes stay in contact with the BM and pericytes in the subendothelial spaces in a phenomenon known as abluminal crawling. Then, leukocytes must breach the vessel wall to exit the graft tissue fully (11). Although the mechanism by which leukocytes breach the BM and reach the graft tissue is not fully characterized, recent evidence from *in vitro* studies suggests a role for FA (attachment of ECs to BM) and pericytes as a barrier for immune invasion (42, 43). We used some common markers to identify pericytes (**Figure 4B**).

We evaluated the (L-R) interaction of T-cells and pericytes with ECs in both borderline and TCMR samples. We used the SingleCellSignalR (44), which links ligands to their receptors in cluster pairs. We did not find major gene difference between borderline and TCMR when we performed DEG (**Supplementary Table 6**). However, L-R analysis between pericytes and ECs reveals different interactions when comparing borderline to TCMR (**Figures 4C, D**) (**Supplementary Tables 7, 8**). In the borderline sample (**Figure 4E**), increase NOTCH4 response to different pericyte ligands such as JAG1, PSEN1, HLA-C, and DLL. The JAG1/NOTCH4 interaction has a role in endothelial maturation (45). Also, NOTCH has a role in vessel stability (46). Moreover, ANGPT1/2 from pericytes interacts with TIE1 in the borderline sample. ANGPT interaction with Tie1 is not well characterized, but it increases vascular stability and angiogenesis (47). Hence, these interactions suggest a possible role of pericytes in controlling ECs stability in the borderline sample. On the other hand, in TCMR (**Figure 4F**), pericytes interact with KDR on ECs, which is not observed in the borderline rejection. The two primary receptors of VEGF, VEGFR1(flt-1) and VEGFR2 (KDR), are receptors for tyrosine kinases (RTKs). KDR is the VEGF activity’s essential meditator, promoting angiogenesis, mitogenesis, and increased permeability (48, 49). In the absence of stability signals and the appearance of KDR, pericytes in the TCMR seem to favor endothelial permeability.

ECs are in close contact with ECM, pericytes, and immune cells during rejection. After describing ECs interaction with ECM and pericytes, we describe EC-T-cell interaction in TCMR and borderline samples using L-R analysis (**Figures 4E, F**) (**Supplementary Tables 7, 8**). In both borderline and TCMR, T-cells expressed IFN-gamma response and allograft rejection genes (**Figure 2A**). However, the interaction between T-cells and ECs was significantly different. In the borderline sample, the T-cells-ECs interaction is characterized by T-cells’ damage-associated molecular patterns (DAMPs), such as HMGB1 and HSP90B, interacting with toll-like receptors 4 (TLR4) on ECs (**Figure 4E**). TLR4, like all TLRs, is mainly expressed on immune cells membrane and is known to induce pro-inflammatory cytokines secretion following DAMPS sensing. However, recently it was demonstrated endothelial cells express major TLRs (50). TLR4 is a receptor for HMGB1 and heat shock proteins (HSPs), which are released in response to cellular stress and injury. The interaction between DAMPs and TLRs is known to activate endothelial cells in an ischemia/reperfusion model (51), but it has not been studied in the context of cellular rejection. On the other hand, in TCMR, many ligands on the immune cells interact with integrins (ITGA5, ITGA2, ITGB3, ITGA8) on ECs, but DAMPS-TLRs interaction was not seen (**Figure 4F**). The latter observations suggest that rejecting T-cells are activating ECs in borderline.

## 4 Discussion

To the best of our knowledge, this is the first study to investigate a borderline change sample from a human kidney allograft at the single-cell resolution. In 2018, Wu and colleagues (2) described the first scRNA seq analysis of a mixed rejection human kidney specimen, suggesting the feasibility of this technique in investigating kidney allografts. Lake and colleagues (20), using snRNA seq, identified human kidney tissue cell populations and molecular diversity. Moreover, in the last years, the single-transcriptome technique was implemented to extend the human kidney atlas (3) and study different kidney diseases (52, 53). Although gene expression of human kidney allografts has been studied extensively using bulk RNA sequencing, this approach has significant limitations. As we and Wu et al (2) demonstrated, most bulk RNA transcripts associated with endothelial cells in rejection were not expressed by ECs. Hence, the bulk analysis could be misleading in reflecting the molecular profile of different cell populations in rejection.

In our case, the T-cell clusters in borderline showed enrichment for allograft rejection and IFN-gamma response pathways (**Figure 2B**); this indicates that T cells are actively involved in rejection in borderline by a high IFN-gamma environment. Nevertheless, EC clusters showed down-regulation of the IFN-gamma signaling pathway in non-rejection and TCMR compared to borderline. IFN-gamma is secreted mainly by activated T-cells (54), NK cells (55), and γδ T cells (56) and is essential in T-cell maturation and cytotoxicity (32). In contrast to expectations, it has been shown that IFN-gamma has a protective role in the early rejection of vascularized allografts (57, 58) but can aggravate vascular injury later in the rejection process (59, 60). Halloran and colleagues demonstrated the protective role of IFN-gamma in heart and kidney rejection in mouse model (58). They showed that IFN-gamma protects against early microcirculation necrosis (58). In our TCMR sample, the down-regulation of IFN-gamma response in ECs possibly reflects negative feedback against excessive ongoing immune invasion or a mechanism that facilitated it, or it could indicate that peak expression of IFN-gamma-associated genes was surpassed.

In the borderline sample, the top DEGs in ECs are not known for their role in the rejection. MALAT1 is a long noncoding RNA that showed a role in endothelial proliferation and migration (61), but its role in rejection is not clear. XAF1, a tumor suppressor gene, has shown an anti-angiogenic effect (62), suggesting the delicate regulation of angiogenesis in the borderline sample. EMCN is a mucin-like sialoglycoprotein that interferes with the assembly of focal adhesion complexes and inhibits the interaction between cells and the extracellular matrix (63). Whether the early up-regulation of EMCN gene is responsible for the later down-regulation of FA pathway in TCMR, and hence facilitating the immune invasion of the graft, needs further investigation.

We found different enriched pathways in EC clusters in different samples. Among the top pathways in ECs of borderline and normal samples were the integrin signaling pathway (FA pathway), VEGF signaling, and angiogenesis. In the case of TCMR, other pathways, such as the endothelin signaling pathway, ranked at the top, and gene-level expression showed that FA genes were less expressed in ECs of the TCMR sample. FA corresponds to the interaction between ITGs in the endothelial membrane with ECM, such as fibronectin (**Figure 4A**). ECM interacts with the endothelium on many levels. First, it represents physical support for ECs on their BM. Second, data showed that ECM interaction with ITGs on ECs controls EC migration, invasion, proliferation, and survival (64–67). Moreover, *in vitro* experiments on T-cells and neutrophils showed that FA forms an obstacle for leukocytes’ subendothelial crawling and breaching the BM (42, 43). Hence, the downregulation of FA genes in our TCMR sample might be facilitating the immune invasion into the graft tissue.

Pericytes, defined as cells in contact with the endothelium and embedded in the vascular BM (67), play an essential role in supporting and influencing ECs (41). In addition to EC-ECM interaction, EC-pericytes constitute another obstacle for immune cells migration through the vessel (36). In the borderline sample, pericytes secrete mediators that interact with endothelial molecules known to participate in endothelial angiogenesis and stabilization (45–47) (**Figure 4C**).

The interaction between T-cells’ DAMPS and ECs’ TLR4 suggests a novel mechanism in cellular rejection. Blocking the TLR4 signaling pathway in a mouse model protected from minor allograft rejection of skin transplant and is associated with reduction of dendritic cell numbers in draining lymph nodes (68). In addition, transcripts of TLR4 and its ligands were found to be upregulated early after transplantation of allogeneic islets (69). HMGB1 also contributes to early rejection failure, as seen in syngeneic islet transplants (70). Herein, the suggested mechanism by which TLRs contribute to the rejection is related to its expression on mononuclear cells. Therefore, cell injury releases DAMPs that activate mononuclear cells expressing TLR leading to cytokine production and recruitment of alloimmune response (71). Nevertheless, the role of TLRs expressed on ECs in cellular rejection is not known. The role of TLR4 expressed on ECs was investigated in an ischemia/reperfusion (I/R) mouse model, where authors demonstrated a novel role of TLR4 in inducing adhesion molecules in ECs (51). In addition, they showed that TLR4 mRNA abundance peaks at 4h following reperfusion and then down-trend (51). Analyzing I/R scRNA-seq mice data set showed that TLR4 expression in ECs starts increasing at 4 h and peaks at 12 h, then downtrend to normal (72), suggesting a role for TLR4 in the early disease state. These observations in I/R models seem to also be valid in our cellular rejection model. T-cells appear to secrete DAMPs that interact with TLR4 on ECs during early rejection, as seen in the borderline sample, but not in a more progressive rejection state (TCMR). Whether this interaction, among other factors, is responsible for the different endothelial phenotypes seen in borderline and TCMR needs validation.

The importance of this data is that it provides a snapshot of what is happening in human allograft during rejection, including all the complicated interactions and influences generated by different cells of the intragraft microenvironment. This is helpful in generating interesting observations and hypotheses and gives insight into the protein expression level (73). The limitations of this study include the following. First, the generalization of the results is limited by the absence of biological replicates. Therefore, the results we found could be confounded by demographic, clinical, and biopsy time differences between the three patients, as indicated in (**Supplementary Table 1**). Second, the L-R interaction analysis is considered an inference on possible interaction and is limited by the number of cells available in each cluster. Third, more validation is needed to verify the upregulation of the FA pathway, EMNC, and TLR4 genes in borderline rejection on the protein level, knowing that finding an *in vitro* model that replicates the complexity of the intragraft microenvironment is challenging and can mask important observations found in our data.

SnRNA-seq is a novel tool for studying cellular transcriptional profiles at a single-cell level, permitting the generation of novel observations and hypotheses. This paper investigated the difference between borderline change sample and TCMR in terms of DEGs, enriched pathways, and interactions between different cell types. We demonstrated that the borderline sample shows a rejection profile at the immune cell level suggesting an early rejection state. Moreover, in our case, ECs appeared to be actively involved in the early rejection process by (a) increasing its response to IFN-gamma stimulation, (b) upregulating the expression of the FA pathway, and (c) expressing TLR4 that interacts with T-cell DAMPs. Hence, we suggest that ECs are actively implicated in the early phases of cellular rejection, and intervening at this level could prevent the progression of the rejection.

## Data availability statement

The data presented in the study are deposited in the NCBI GEO repository, accession number GSE228300.

## Ethics statement

The studies involving human participants were reviewed and approved by MASS GENERAL BRIGHAM IRB. The patients/participants provided their written informed consent to participate in this study.

## Author contributions

AH performed most the scientific analysis and literature review. AH and AE wrote the first draft of the manuscript. AH and AE performed the biostatistical analysis under the supervision of PK, AG, and JA. KV and ZS processed the samples. AS, NY, CD, MM, JC and RE helped with some of the analysis and literature review. AW, LR, SD, RA, and PC helped review and edit the manuscript. JA and AG planned the study design. All authors contributed to the article and approved the submitted version.
