# Supplementary material for: Uncovering a novel role of focal adhesion and interferon-gamma in cellular rejection of kidney allografts at single cell resolution
Source: Front Immunol. 2023 Mar 31;14:1139358. doi: 10.3389/fimmu.2023.1139358 (PMC10102512; doi:10.3389/fimmu.2023.1139358)
Supplement: Supplementary file 1 [file Table_1.docx]

Table 1 Clinical characteristics of donors and recipients

| **Clinical information** | **Kidney Biopsies** | | |
| --- | --- | --- | --- |
|  | **Non-rejection** | **Borderline** | **TCMR** |
| **Donor** | Living | Deceased | Living |
| Living/Deceased |  |  |  |
| Age at donation | 66 | 47 | 67 |
| Gender | F | M | F |
| CMV  EBV | +  + | -  + | -  - |
| **Recipient** |  |  |  |
| Year/Age at transplantation | 12/27/2018(75) | 10/8/2016(60) | 2/13/2018(43) |
| Gender | M | M | M |
| Race/Ethnicity | African-American | White | African-American |
| Native kidney disease | Renal Cell Carcinoma/ Hypertension | Hypertension | Diabetes mellitus type 1 |
| Nb of Transplant | 1 | 1 | 2 |
| CMV  EBV | -  + | +  + | +  + |
| **At the time of transplantation** | A(1 MM), B(1MM), DR(1MM) | A(2MM), B(2MM), DR(1MM | A(2MM), B(2MM), DR(1MM) |
| HLA-ABDR miss match (MM) |  |  |  |
| DSA | None | None | None |
| Induction immunosuppression | Basiliximab | Basiliximab | Basiliximab |
| Maintenance immunosuppression (at the time of biopsy) | -Mycophenolate sodium  -Prednisone  -Tacrolimus | Mycophenolate sodium  -Prednisone  -Belatacept | -Everolimus  -Tacrolimus  -Prednisone |
| **Index biopsy used for snRNA-seq** | 4/4/2019 | 6/6/2019 | 5/16/2019 |
| Serum creatinine, mg/dl(time of bx) | 3.17 | 2.99 | 3.97 |
| Index biopsy Banff lesion scores | Non-rejection | Borderline | TCMR Banff IIa |
