# Supplementary figures and images for: Uncovering a novel role of focal adhesion and interferon-gamma in cellular rejection of kidney allografts at single cell resolution
Source: Front Immunol. 2023 Mar 31;14:1139358. doi: 10.3389/fimmu.2023.1139358 (PMC10102512; doi:10.3389/fimmu.2023.1139358)

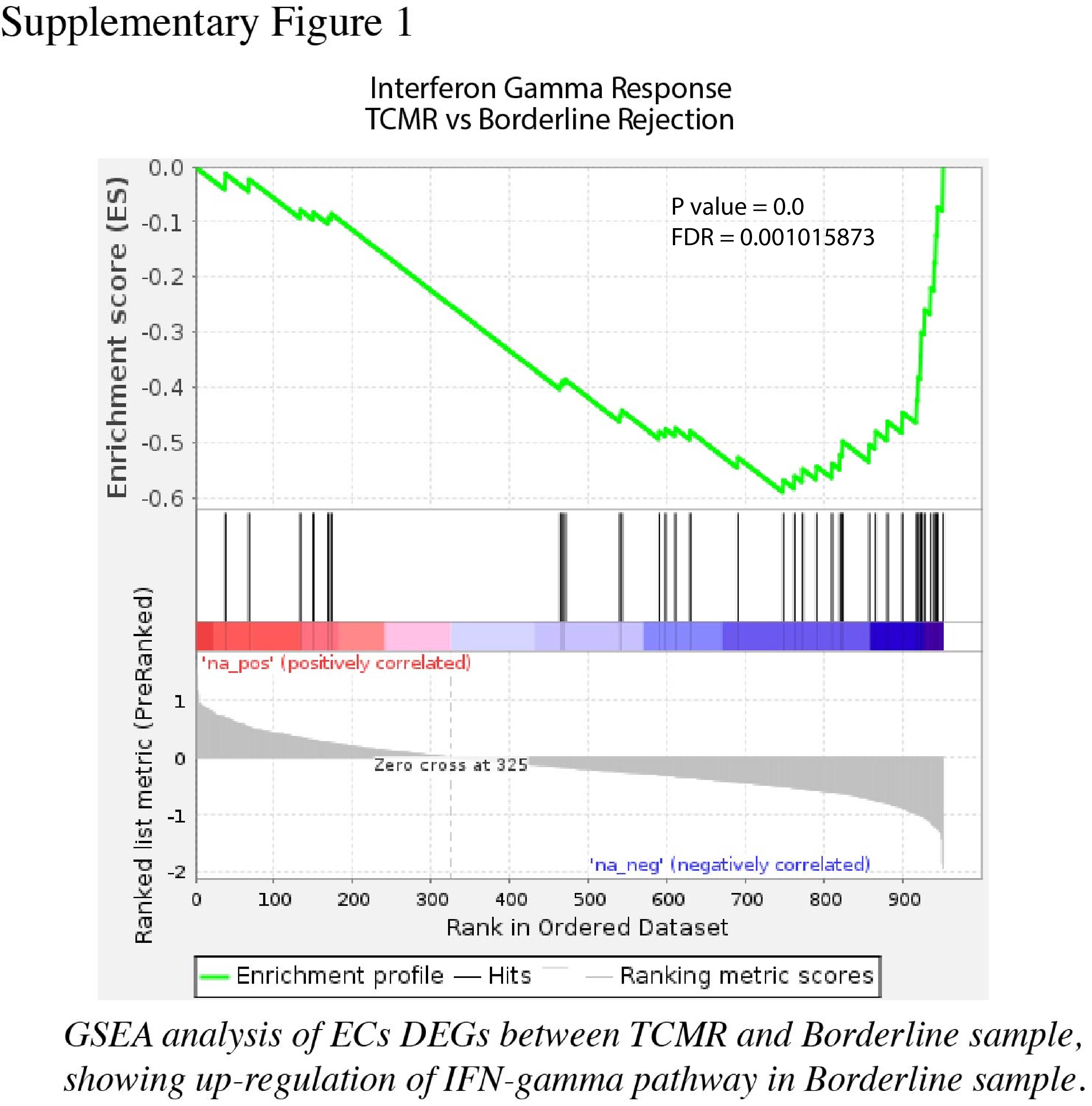

Supplement: Supplementary file 9 [file Image_1.jpeg]
